# Dosimetric predictors of temporal lobe injury after intensity-modulated radiotherapy for T4 nasopharyngeal carcinoma: a competing risk study

**DOI:** 10.1186/s13014-019-1229-9

**Published:** 2019-02-08

**Authors:** Juan Huang, Fang-fang Kong, Ronald Wihal Oei, Rui-ping Zhai, Chao-su Hu, Hong-mei Ying

**Affiliations:** 1 Department of Radiation Oncology, Fudan University Shanghai Cancer Center, Shanghai, China; 2 Department of Oncology, Shanghai Medical College, Fudan University, Shanghai, China

## Abstract

**Background:**

In patients with T4 nasopharyngeal carcinoma (NPC), death may occur prior to the occurrence of temporal lobe injury (TLI). Because such competing risk death precludes the occurrence of TLI and thus the competing risk analysis should be applied to TLI research. The aim was to investigate the incidence and predictive factors of TLI after intensity-modulated radiotherapy (IMRT) among T4 NPC patients.

**Methods:**

From March 2008 to December 2014, T4 NPC patients treated with full-course radical IMRT at our center were reviewed retrospectively. A nested case-control study was designed for this cohort of patients. The cases were patients with TLI diagnosed by MRI during the follow-up period, and the controls were patients without TLI after IMRT matched 1:1 to each case by gender, age at diagnosis, intercranial involvement, and follow-up time. The end point was time to TLI or death without prior TLI. We analyzed the cumulative incidence function (CIF) and performed a competing risk regression model to identify the predictors of TLI.

**Results:**

With a median follow-up of 40.1 months, 63 patients (63/506, 12.5%) developed TLI as diagnosed by MRI, and 136 deaths occurred during the period. The cumulative incidence of TLI at 5 years was 13.2%, while 26.7% died without prior TLI. The univariate analysis showed that all selected dosimetric parameters were associated with the occurrence of TLI. On multivariate analysis, D1cc and V20 remained statistically significant. Based on the area-under-the-curve (AUC) values, D1cc was considered the most predictive. The patients with D1cc > 71.14 Gy had a 7.920-fold increased risk of TLI compared with those with D1cc ≤71.14 Gy (*P* < 0.05). Similarly, V20 > 42.22 cc was found to result in a statistically significant higher risk of TLI (subdistribution hazard ratio [sHR] =3.123, *P* < 0.05).

**Conclusions:**

TL D1cc and V20 were predictive of TLI after IMRT for T4 NPC. They should be considered as first and second priorities of dose constraints of the TL. D1cc ≤71.14 Gy and V20 ≤ 42.22 cc could be useful dose-volume constraints for reducing the occurrence of TLI during IMRT treatment planning without obviously compromising the tumor coverage.

## Background

Despite its rarity in most parts of the world, nasopharyngeal carcinoma (NPC) is a very common endemic disease in southeast Asia and eastern Asia [1]. Due to its anatomic location and radio-sensitivity, radiotherapy is the primary modality of treatment for non-distant metastatic NPC. Compared with conventional techniques, intensity-modulated radiotherapy (IMRT) provides better loco-regional control and normal tissue sparing [2–8]. However, late toxicities remain a problem. Temporal lobe injury (TLI) after IMRT is one of the most serious complications; TLI not only causes clinical symptoms such as headache, dizziness, neurocognitive dysfunctions but also adversely affects patient quality of life [9, 10]. IMRT was expected to reduce TLI occurrence. However, patients with early-stage disease benefit more from IMRT, and the incidence of TLI does not appear to be reduced in T4 patients [11]. T4 NPC often infiltrates the skull base or invades intracranial tissues. The temporal lobe (TL) is so close to the tumor that it is partially included in the radiation treatment fields and is inevitably exposed to high-dose radiation. Currently, the exact incidence, especially the actuarial rate of this late complication in T4 NPC, has not been well reported, and few data are available.

In the past few years, several studies about TLI after IMRT have been published [12–20]. However, a dose-volume relationship, including a definite threshold dose for the TL, remains unclear. Currently, the TL dose constraint recommended by the Radiation Therapy Oncology Group (RTOG) is maximum dose (Dmax) ≤60 Gy or the volume receiving 65 Gy (V65) ≤1%. However, these constraints seem to be conservative and make it difficult to provide dose coverage to the tumor in T4 NPC. How to achieve an optimal balance between TL protection and tumor coverage needs to be addressed. Due to technological advances, dosimetric parameters based on a dose-volume-histogram (DVH) generated from IMRT plans make it possible to analyze the relationship between TLI and the actual dose distribution in the TL. Moreover, the efficacy and acute toxicities for the improved chemotherapy regimens and new targeted biologic agents have been assessed in patients with locally advanced NPC; however, data on late toxicities are lacking.

Patients with T4 NPC often represent a multimorbid population with an increased risk of death resulting from NPC or other causes. The occurrence of death hinders the occurrence of TLI. A particular situation emerges when interest is focused on TLI in the presence of death, which alter the probability of TLI. This is the case of competing risk events. Although the appropriate use of competing risk methodology has been advocated in other diseases [21], the competing risk analysis has not been applied to TLI research to date. In this study, we used a competing risk analysis to estimate the incidence of TLI and determine the clinical and dosimetric factors that could predict the risk of TLI occurrence. Furthermore, we aimed to identify useful dose constraints to guide treatment planning for patients with T4 NPC based on our own experience.

## Methods

### Patient population and study design

This retrospective study was approved by the institutional review board of our cancer center. Written informed consent was obtained from each patient before treatment. From March 2008 to December 2014, previously untreated, pathologically confirmed, non-distant metastatic NPC patients treated with full-course radical IMRT at our center were reviewed. All patients were re-staged according to the American Joint Committee on Cancer/AJCC 2010 staging system. Patients with T4 NPC who received head and neck MRI after IMRT with a follow-up interval of more than six months were included. Patients with re-irradiation to the head and neck or coexisting malignancies were excluded. A nested case-control study was designed for this cohort of patients. The case group included T4 NPC patients with TLI diagnosed by MRI during the follow-up period, and the control group included patients without TLI after IMRT who were matched 1:1 to each case by gender, age at diagnosis (±2 years), intercranial involvement, and follow-up time. The diagnosis date of each case represented the index date of this matched case-control study. The eligible controls did not have a diagnosis of TLI prior to the index date. The clinical information was reviewed retrospectively from a clinical database at our center.

### IMRT

The target volumes were delineated according to the recommendations of the International Commission on Radiation Units and Measurements Reports 50 and 62. The gross tumor volume (GTV) included the primary nasopharyngeal tumor or enlarged retropharyngeal nodes (GTV_NX) and positive neck lymph nodes (GTV_LN). The planning target volume (PTV) for the GTV (PTV_G) was defined by adding a uniform 5-mm margin to the GTV. Two clinical target volumes (CTVs) were delineated at our center. The CTV1 was defined as the high-risk regions that encompassed PTV_G with a margin of no less than 3 mm and included the entire nasopharynx, retropharyngeal lymph nodal regions, clivus, skull base, parapharyngeal space, pterygopalatine fossae, entire sphenoid sinus, posterior third of the nasal cavity and maxillary sinuses, and high-risk nodal regions. The CTV2 was defined as the low-risk lymph nodal regions that were not included in the CTV1. The PTV for the CTV (PTV_C) was defined as the CTV with a 3-mm margin in each direction. When the CTV was close to critical organs, such as the brainstem and spinal cord, the margin was minimized to 1 mm. The critical organs at risk (OARs), including the brainstem, spinal cord, optic chiasm, optic nerves, eyeballs, lenses, temporomandibular joints, mandible, TLs, oral cavity, larynx and parotid glands, were contoured before IMRT optimization. The dose constraints of the OARs were as follows: the maximum dose of the brainstem, optic chiasm and nerves should be ≤54 Gy (allowing 1% volume < 60 Gy), spinal cord ≤45 Gy (allowing 1 cm^3^ spinal cord < 50 Gy), and lens ≤8 Gy. Efforts were exerted to restrict the mean dose of the parotid glands and the maximum dose of the TLs as much as possible without compromising the dose coverage to the tumor.

A nine-field IMRT plan with equally spaced gantry angles delivered by step-and-shoot and simultaneous integrated boost (SIB) techniques with 6-MV photon beam of a linear accelerator was created by Pinnacle (Pinnacle 3, Philips Corp., Fitchburg, WI) Treatment Planning System (TPS). The prescribed dose was 70.4 Gy to PTV_G, 66 Gy to PTV_LN, 60 Gy to PTV_C at high risk, and 54 Gy to PTV_C at low risk in 32 fractions. Residual disease was treated by external beam IMRT or brachytherapy.

### Chemotherapy

Following our institutional protocols, chemotherapy was a standard treatment for locoregionally advanced (stages III-IV) NPC patients without serious medical comorbidities. The commonly used chemotherapy modalities included induction chemotherapy plus concurrent chemo-radiotherapy, induction chemotherapy plus adjuvant chemotherapy and concurrent chemo-radiotherapy plus adjuvant chemotherapy. Generally, induction chemotherapy was used in patients whose waiting time for RT was too long or patients with bulky tumors needed to be downsized. Adjuvant chemotherapy was applied when residual lesions existed after radiotherapy. Most hospitalized patients received induction chemotherapy plus concurrent chemo-radiotherapy. As a part of the clinical trial, most outpatients received induction chemotherapy plus adjuvant chemotherapy. Based on prior treatment assessment, a few patients received concurrent chemo-radiotherapy plus adjuvant chemotherapy. The common regimens of induction or adjuvant chemotherapy included TPF (docetaxel, 60 mg/m^2^/day, day 1, cisplatin, 25 mg/m^2^/day, days 1–3, and 5-fluorouracil [5-FU], 500 mg/m^2^/day, days 1–3), TP (docetaxel, 60 mg/m^2^/day, day 1, and cisplatin, 25 mg/m^2^/day, days 1–3), PF (cisplatin, 25 mg/m^2^/day, days 1–3, and 5-FU, 500 mg/m^2^/day, days 1–3), and GP (gemcitabine, 1000 mg/m^2^/day, day 1 and day 8, and cisplatin, 25 mg/m^2^/day, days 1–3). Induction chemotherapy was administered every 3 weeks before IMRT, and adjuvant chemotherapy was applied every 3 weeks beginning 4 weeks after the completion of IMRT. Concurrent chemotherapy consisting of cisplatin was used weekly (40 mg/m^2^) or every 3 weeks (80 mg/m^2^). In addition to chemotherapy and IMRT, 36 patients received concurrent cetuximab (initial dose: 400 mg/m^2^, followed by 250 mg/m^2^ weekly) or nimotuzumab (200 mg weekly).

### Follow-up and diagnostic criteria for TLI

Follow-up MRI of the head and neck was performed 3 months after IMRT, then once every six months for 5 years and annually after that. The end point in this analysis was time from the first day after the completion of IMRT to the date of TLI occurrence or death (i.e., death without prior TLI was designated “prior death”). In competing risk terms, TLI corresponds to the event of interest, and the competing risk event is prior death. The TLI latency was calculated from the first day after the completion of IMRT to the date of the first MRI indicating the presence of TLI. TLI was diagnosed based on MRI images that were independently reviewed by two experienced radiologists and one oncologist. Other intracranial diseases, tumor recurrence and metastasis were excluded before the diagnosis. The diagnostic criteria were as follows: 1) white matter lesions: finger-like lesions with a high signal intensity on T2-weighted images (T2WIs); 2) contrast-enhanced lesions: enhanced nodules with or without necrosis on post-contrast T1-weighted images (T1WIs); and 3) cysts: round or oval lesions with a high signal intensity on T2WIs with a thin or imperceptible wall [22].

### TL re-contouring and dosimetric parameters collection

Original IMRT plans of the case and control groups were restored to the TPS. TLs were re-contoured by the same experienced oncologist according to a recommended contouring method [23]. DVH-based dosimetric parameters for each TL were collected, including Dmax, D1cc (the maximum dose to 1 ml of the TL), and Vx (absolute volumes of the TL receiving at least x Gy; the values of x were 20, 30, 40, 50, 60, and 70).

### Statistical analysis

The overall survival rate was calculated using the Kaplan-Meier method. A competing risk analysis was performed. The cumulative incidence function (CIF), which addresses competing events, was calculated to estimate the cumulative incidence of TLI in the presence of death. Gray’s test was performed to compare the equality of CIFs in subgroups. A Fine-Gray competing risk regression model for subdistribution hazard ratio (sHR) was employed to analyze the effect of the predictors on the development of TLI. The dosimetric parameters were compared between the injured and healthy TLs with an independent sample t-test, and the categorical variables were compared by a chi-square test or Fisher’s exact test. The candidate variables included N-classification, overall stage, overall treatment time (OTT), the use of induction chemotherapy, adjuvant chemotherapy, concurrent chemotherapy, concurrent cetuximab or nimotuzumab, nasopharynx boost (by external beam IMRT) and multiple dosimetric parameters. Univariate and multivariate competing risk regression models were created to determine the clinical and dosimetric factors associated with the development of TLI. Receiver operating characteristic (ROC) curve analyses were conducted to identify the optimal cut-off values of the dosimetric parameters obtained in the final multivariate model. ROC and area-under-the-curve (AUC) values were compared using the the methods described by DeLong et al. to determine the best predictor of TLI. All statistical analyses were performed using Statistical Product and Service Solutions (SPSS) software version 22.0 (IBM Corporation, Armonk, NY, USA) and R version 3.5.2 with the cmprsk, crr and pROC package. All confidence intervals (CIs) are reported at the 95% confidence level. Any result with a two-sided *P*-value < 0.05 was considered statistically significant.

## Results

From March 2008 to December 2014, 506 patients fulfilled the inclusion criteria. The age range was 16–81 years old (median, 48 y), and the male to female ratio was 3.56:1. Baseline characteristics are shown in Table 1. The median follow-up time was 40.1 months, with a range from 6 months to 120.1 months. The overall survival at 1, 2, 3, 4 and 5 years was 99.6, 93.9, 84.4, 79.4, and 71.9%, respectively.

**Table 1:** Baseline Characteristics

Variable	Number (%)
Age range (y)	16–81
Median age (y)	48
Male	395 (78.1)
Female	111 (21.9)
N-classification	
N0	55 (10.9)
N1	194 (38.3)
N2	198 (39.1)
N3	59 (11.7)
AJCC 2010 stage	
IVA	447 (88.3)
IVB	59 (11.7)
Chemotherapy	
Induction	467 (92.4)
Concurrent	292 (57.7)
Adjuvant	128 (25.3)
Concurrent cetuximab or nimotuzumab	36 (7.1)
Nasopharynx boost (by external IMRT)	23 (4.6)
Overall treatment time OTT)	
≤ 45	193 (38.1)
> 45	313 (61.9)

*Abbreviation*: *AJCC* American Joint Committee on Cancer

### Cumulative incidence of TLI and prior death

**Fig. 1:**
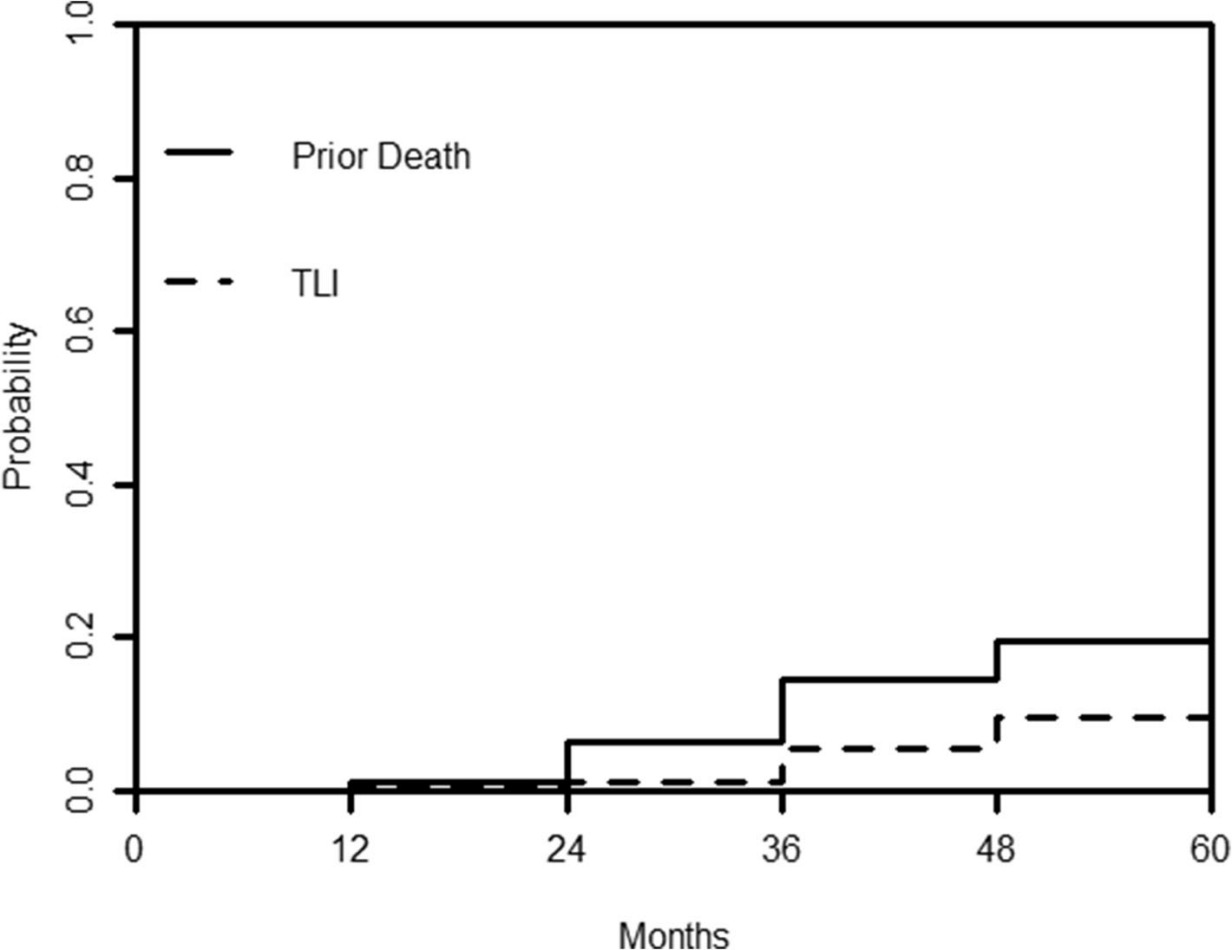
Estimated cumulative incidence curves with death prior to TLI and TLI as competing events in 506 patients with T4 NPC after IMRT. TLI = temporal lobe injury; NPC = nasopharyngeal carcinoma; IMRT = intensity-modulated radiotherapy

With a median follow-up time of 40.1 months, 63 patients (63/506, 12.5%) developed TLI as diagnosed by MRI, and 136 patients died during the period. In the presence of death as a competing risk event, the estimated cumulative incidence of TLI from 1 to 5 years was 0.4, 1.1, 5.4, 9.6, and 13.2%. Whereas, the estimated 5-year probability of death without prior TLI was 26.7% (Fig. 1).

**Fig. 2:**
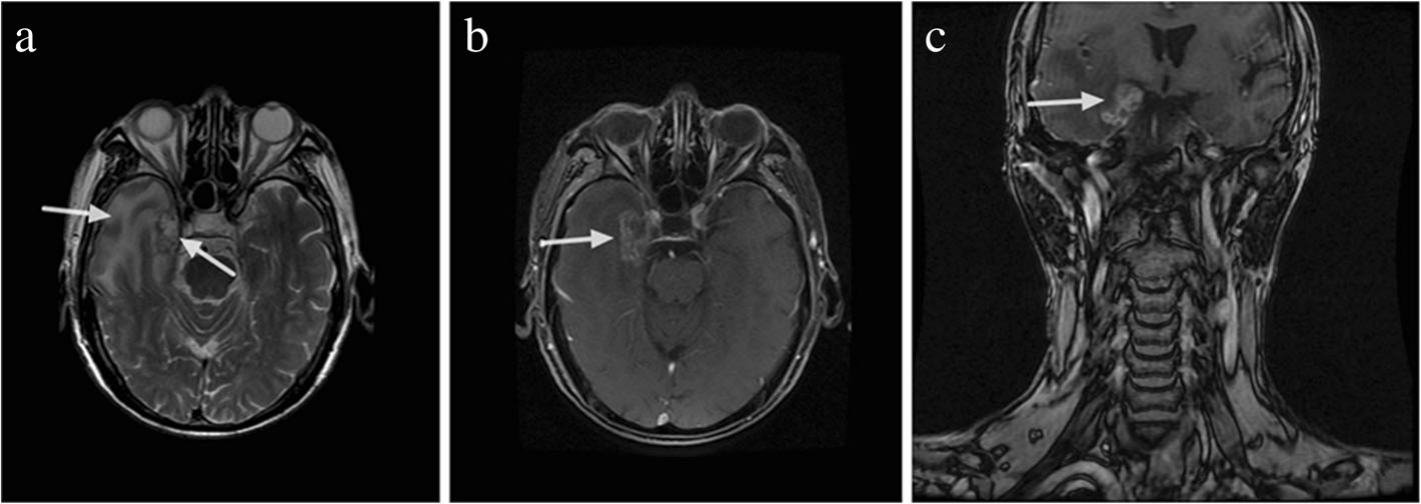
An example of temporal lobe injury showing (**a**) white matter lesions with a high signal intensity on T2-weighted images, (**b**) contrast-enhanced lesions on post-contrast T1-weighted images, and (**c**) lesions on the sagittal MR images

Among the 63 patients with TLI, 43 patients (68.25%) had unilateral TLI, and 20 patients (31.75%) had bilateral TLI. Thus, a total of 83 injured TLs were detected. Twenty-nine patients (46.03%) had clinical symptoms, including headache, dizziness, memory impairment, epileptic attacks, and changes in personality and consciousness. According to Common Terminology Criteria for Adverse Events version 4.0, 21 patients (72.41%) presented grade 1 TLI, 5 patients (17.24%) presented grade 2 TLI and 3 patients (10.34%) presented grade 3 TLI. Figure 2 shows a typical MRI of a patient with TLI.

### Clinical and dosimetric factors associated with TLI occurrence

Of the 506 patients recruited for the study, sixty-three patients with TLI and sixty-three patients without TLI were matched 1:1 by gender, age at diagnosis, intercranial involvement, and follow-up time. The patient and treatment characteristics of the case and control groups are summarized in Table 2. Univariate analyses of 126 patients showed that N-classification, overall stage, OTT, the use of induction chemotherapy, adjuvant chemotherapy, concurrent chemotherapy, concurrent cetuximab or nimotuzumab, and nasopharynx boost (by external beam IMRT) were not associated with the development of TLI (*P* > 0.05). However, all selected dosimetric parameters of the 83 injured and 169 healthy TLs in the univariate analyses were found to be associated with the occurrence of TLI (*P* < 0.001). The results are shown in Table 3.

**Table 2:** Patient and treatment characteristics of the case group and control group

Variable	Case group	Control group	*P* value
Median age (y)	49	48	ND
Age range (y)	27–72	28–70	ND
Male	56 (88.9)	56 (88.9)	ND
Female	7 (11.1)	7 (11.1)	ND
N-classification			0.069
N0–1	31 (49.2)	41 (65.1)	
N2–3	32 (50.8)	22 (34.9)	
AJCC 2010 stage			0.752
IVA	58 (92.1)	57 (90.5)	
IVB	5 (7.9)	6 (9.5)	
Chemotherapy			
Induction	60 (95.2)	56 (88.9)	0.187
Concurrent	36 (57.1)	42 (66.7)	0.271
Adjuvant	20 (31.7)	19 (30.2)	1.000
Concurrent cetuximab or nimotuzumab	7 (11.1)	3 (4.8)	0.187
Nasopharynx boost	4 (6.3)	1 (1.6)	0.365^a^
Overall treatment time (OTT)			0.859
≤ 45	25 (39.7)	19 (30.1)	
> 45	38 (60.3)	44 (69.8)	
Temporal lobe Dmax (Gy)	75.22 (63.83–80.16)	72.64 (61.35–79.35)	< 0.001
Temporal lobe D1cc (Gy)	72.52 (54.47–77.21)	66.81 (54.40–74.95)	< 0.001
Temporal lobe V20 (cc)	48.18 (21.26–75.20)	38.06 (15.01–79.18)	< 0.001
Temporal lobe V30 (cc)	31.64 (10.22–56.35)	23.94 (8.55–55.09)	< 0.001
Temporal lobe V40 (cc)	20.11 (6.09–43.16)	15.09 (5.63–37.47)	< 0.001
Temporal lobe V50 (cc)	13.23 (2.76–31.28)	8.84 (2.23–29.56)	< 0.001
Temporal lobe V60 (cc)	7.59 (0.2–19.71)	3.65 (0.24–19.45)	< 0.001
Temporal lobe V70 (cc)	2, 26 (0–9.18)	0.26 (0–8.22)	< 0.001

*Abbreviation*: *AJCC* American Joint Committee on Cancer, *ND* not done;

^a^*P* value with Fisher Exact Test

Values are number (percentage) or median (range)

**Table 3:** Estimated subdistribution hazard ratios for temporal lobe injury (TLI) using univariate and multivariate competing risk regression models

Variable	Univariate analyses		Multivariate analyses	
	sHR^a^ (95% CI)	*P* value	sHR^a^ (95% CI)	*P* value
N-classification				
N2–3 vs N0–1	1.330 (0.853–2.070)	0.210		
AJCC 2010 stage				
IVB vs IVA	0.806 (0.265–2.450)	0.700		
Chemotherapy				
Induction (vs no)	2.270 (0.794–6.480)	0.130		
Concurrent (vs no)	0.744 (0.445–1.240)	0.260		
Adjuvant (vs no)	1.080 (0.618–1.890)	0.790		
Concurrent cetuximab or nimotuzumab (vs no)	1.760 (0.803–3.850)	0.160		
Nasopharynx boost (vs no)	2.430 (0.919–6.440)	0.074		
Overall treatment time (OTT)				
> 45 vs ≤45	0.941 (0.567–1.560)	0.810		
Temporal lobe Dmax (per Gy increase)	1.260 (1.150–1.370)	< 0.001		
Temporal lobe D1cc (per Gy increase)	1.270 (1.180–1.370)	< 0.001	1.500 (1.212–1.856)	< 0.001
Temporal lobe V20 (per cc increase)	1.040 (1.030–1.060)	< 0.001	1.072 (1.009–1.139)	0.024
Temporal lobe V30 (per cc increase)	1.060 (1.040–1.090)	< 0.001		
Temporal lobe V40 (per cc increase)	1.100 (1.070–1.120)	< 0.001		
Temporal lobe V50 (per cc increase)	1.130 (1.090–1.170)	< 0.001		
Temporal lobe V60 (per cc increase)	1.190 (1.130–1.250)	< 0.001		
Temporal lobe V70 (per cc increase)	1.380 (1.260–1.510)	< 0.001		

*sHR*^a^ subdistribution hazard radio

**Fig. 3:**
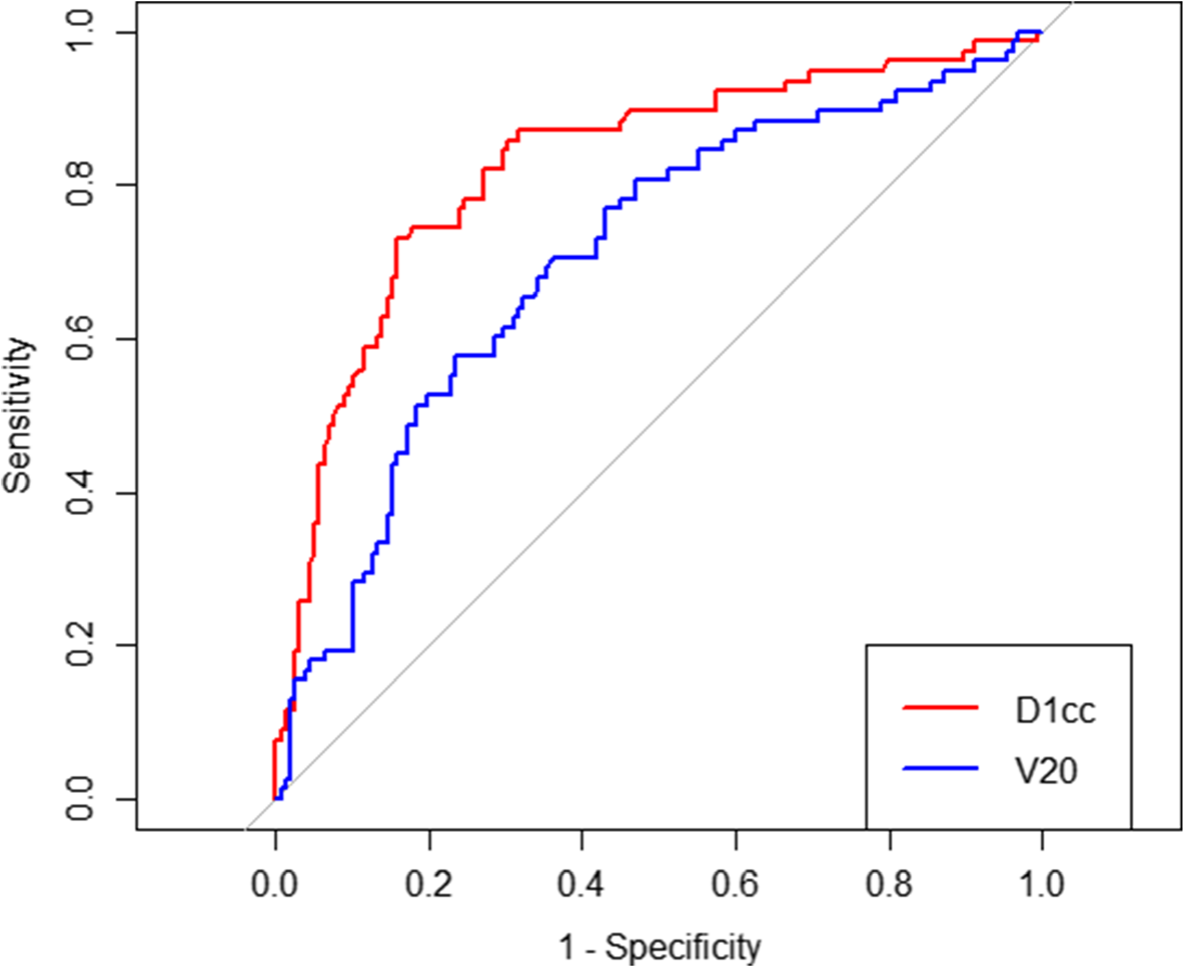
Receiver operating characteristic curves for temporal lobe D1cc and V20

After adjusting for the clinical factors, the Fine-Gray competing risk regression model revealed that TL D1cc and V20 remained statistically significant and were independent predictors of TLI (Table 3). Patients with higher D1cc were more likely to experience TLI (sHR, 1.500 per 1 Gy increase; 95% CI, 1.212 to 1.856; *P* < 0.001). Similarly, patients with larger V20 had a higher risk of TLI (sHR, 1.072 per 1 cc increase; 95% CI, 1.009 to 1.139; *P* < 0.001). ROC curves were generated for D1cc and V20 and the optimal cutoff values were 71.14 Gy and 42.22 cc, respectively (Fig. 3). The AUC value of D1cc was statistically higher than the AUC value of V20 for TLI (0.825 [95% CI 0.765–0.884] versus 0.706 [95% CI 0.635–0.777]; *P* = 0.007) (Fig. 3). Based on the AUC values, D1cc should be considered as the most predictive. After adjusting for the clinical factors, the patients with D1cc > 71.14 Gy had a 7.920-fold increased risk of TLI compared with those with D1cc ≤71.14 Gy (*P* < 0.001). Similarly, V20 > 42.22 cc was found to result in a statistically significant higher risk of TLI (sHR =3.123, *P* < 0.001).

**Fig. 4:**
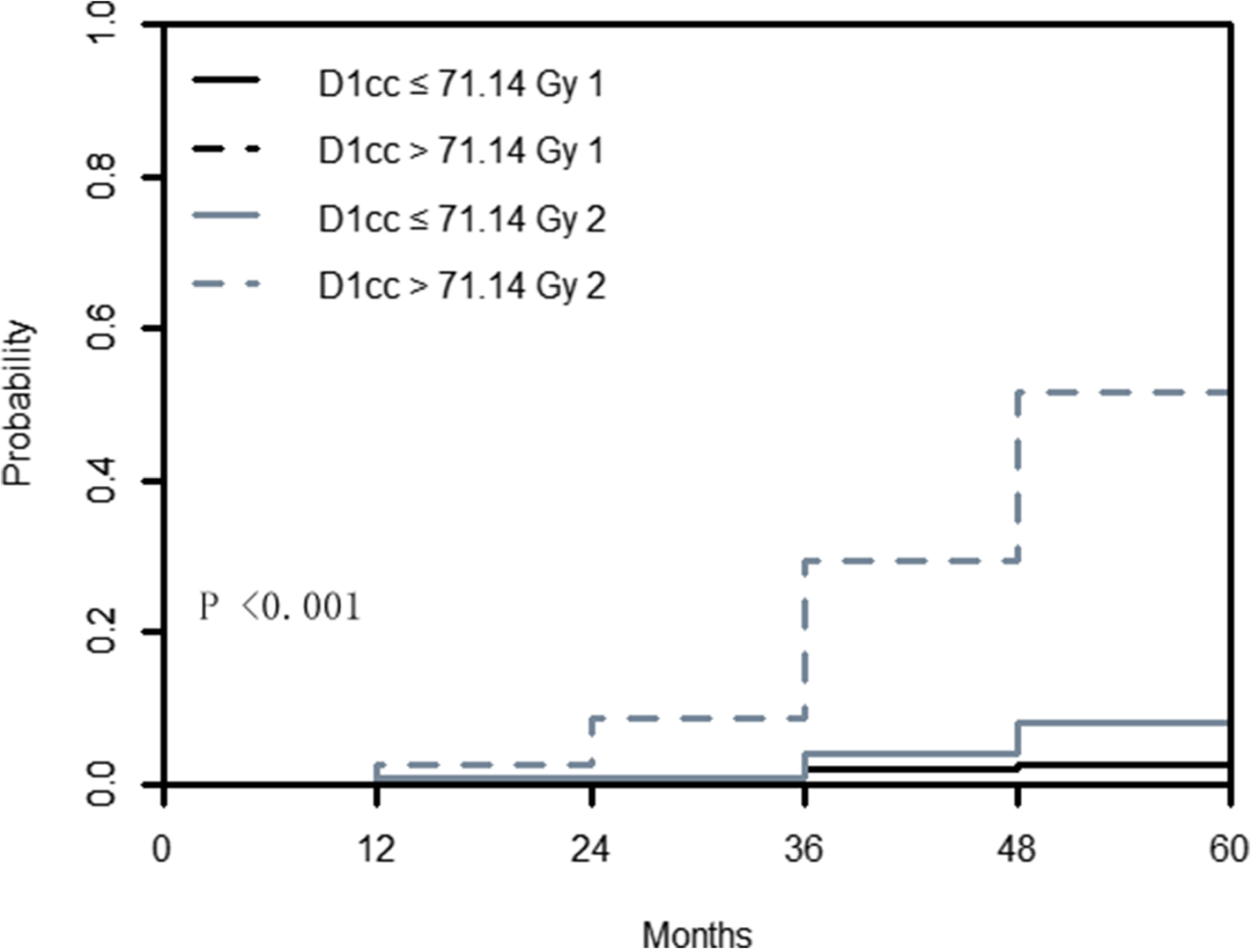
Estimated cumulative incidence curves with death prior to TLI (1) and TLI (2) as competing events for T4 NPC stratified by temporal lobe D1cc ≤71.14 Gy and D1cc > 71.14 Gy. TLI = temporal lobe injury; NPC = nasopharyngeal carcinoma

**Fig. 5:**
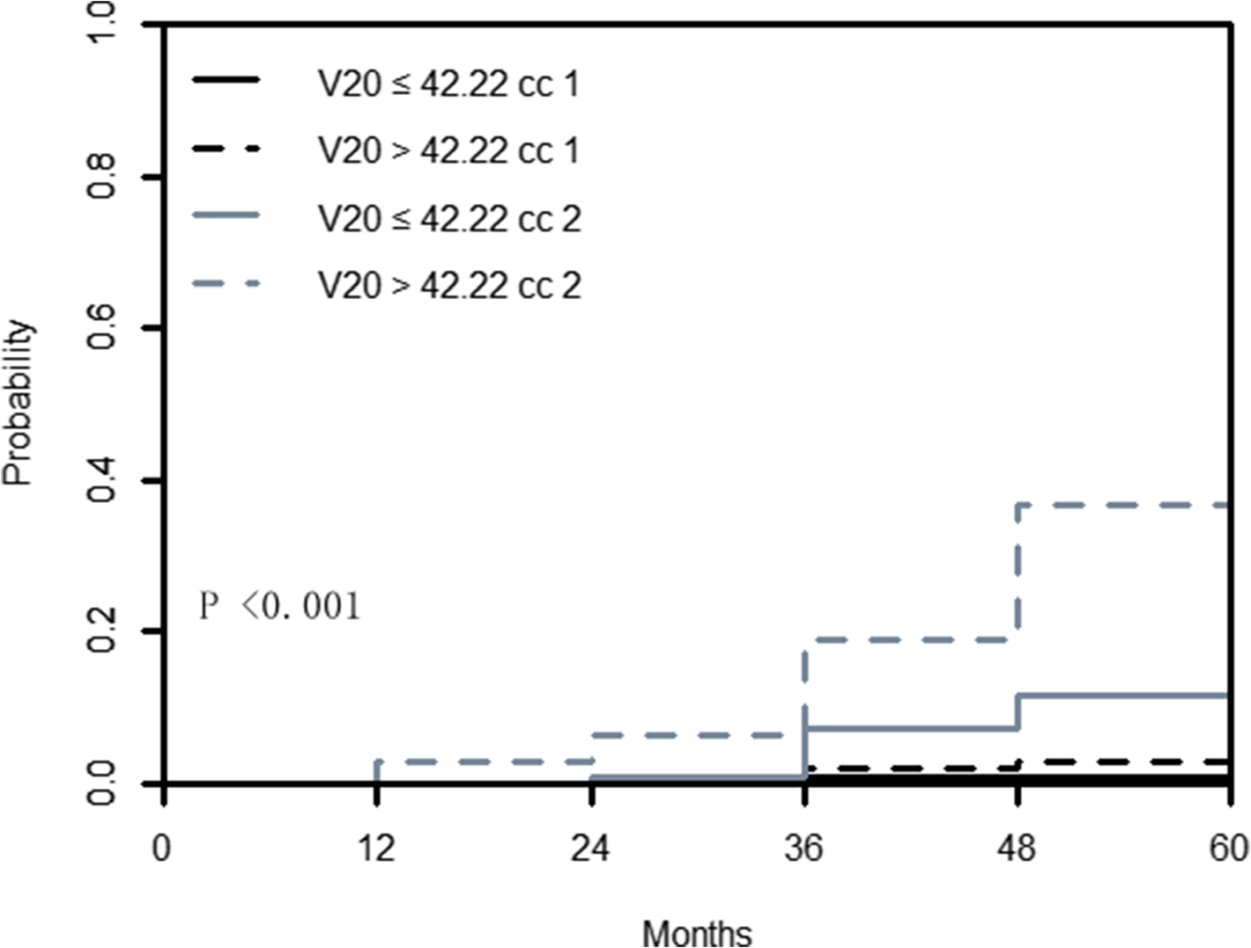
Estimated cumulative incidence curves with death prior to TLI (1) and TLI (2) as competing events for T4 NPC stratified by temporal lobe V20 ≤ 42.22 cc and V20 > 42.22 cc. TLI = temporal lobe injury; NPC = nasopharyngeal carcinoma

In this nested case-control study, Gray’s test further indicated that CIFs for the patients with D1cc ≤71.14 Gy and patients with D1cc > 71.14 Gy were not statistically different for prior death (coded as 1) (*P* = 0.166), but they were highly significant for TLI (coded as 2) (*P* < 0.001). The 5-year cumulative incidence of TLI in the group with D1cc ≤71.14 Gy was 13.2%, which was much lower than the 5-year cumulative incidence of TLI in the group with D1cc > 71.14 Gy (62.2%, *P* < 0.001). A plot of CIFs for TLI and prior death across groups is also produced (Fig. 4). However, CIFs for the group with V20 ≤ 42.22 cc and group with V20 > 42.22 cc were statistically different for prior death (coded as 1) (*P* = 0.024) and TLI (coded as 2) (*P* < 0.001). And the 5-year cumulative incidence of TLI in the patients with V20 ≤ 42.22 cc and V20 > 42.22 cc was 17.9 and 44.1%, respectively (*P* < 0.001) (Fig. 5).

## Discussion

To the best of our knowledge, this is the first study to apply competing risk analysis to TLI research in patients with NPC. Among T4 NPC patients, death may occur prior to the TLI occurrence. Since such competing risk events exclude the event of interest, competing risk events should be taken into account in cumulative incidence calculation or prognostic models. We prefer the competing risk methodology over the Kaplan-Meier method or standard Cox regression model. The frequently used Kaplan-Meier method, in contrast to the CIF, censors patients who die prior to TLI. This leads to overestimated probabilities of TLI because dead subjects (and thus censored) are considered to experience TLI in the future. Similarly, in the Fine and Gray model, the competing events are not censored but are treated as different (competing) events.

### Incidence of TLI after IMRT in patients with T4 NPC

The crude incidence of TLI in patients with T4 NPC has been reported to range from 7.5 to 28% [12, 17, 24–27]. In this study, 63 patients developed TLI with an incidence of 12.5%, which was consistent with previous reports. The differences in the follow-up duration might cause a deviation in the incidence. TLI is a late complication with a long latency; the longer the follow-up period, the greater the possibility of TLI occurrence. Zhou et al. [11] reported that the 5-year cumulative incidence of TLI in patients with NPC after two-dimensional radiotherapy (2D-RT) was 34.9%, which was much higher than the finding in our study (13.2%). In a previous study involving 973 NPC patients after 2D-RT at our center, the 4-year cumulative incidence of TLI was 15.3% (data not published). This finding was higher than that reported in this study (9.6%). On the one hand, these data suggest that IMRT could better protect the TL than 2D-RT in T4 NPC. On the other hand, compared with early-stage NPC patients, the T4 patients had a shorter survival period, and some patients died before TLI occurrence. We calculated the CIF by appropriate accounting. However, the analysis in the paper mentioned above was performed using the Kaplan-Meier method, which censors death as it occurs and over-estimates the CIF in the presence of competing risks. In addition, when comparing the incidence of TLI with older studies, it is worth noting whether an MRI was used as a routine examination to detect TLI during the follow-up and whether the endpoints were the same. Moreover, due to patient loss to follow-up and a relatively short follow-up period, the incidence of TLI might be underestimated, and long-term outcomes should be updated in the future.

### Clinical and dosimetric factors associated with TLI occurrence

Several clinical factors, such as the prescribed total dose, dose per fraction, OTT, chemotherapy, age and T category, have been reported to be associated with TLI occurrence [9, 28–30]. Since our study was a case-control study in which each case was matched to a control by age and gender and all patients had T4 NPC, we could not analyze the differences in age, gender and T category between the two groups. Moreover, due to the standardized treatment modalities for T4 NPC at our center, this study failed to draw such conclusions. The study conducted by Zhou et al. [16], which involved 1883 NPC patients, suggested that the use of concurrent cetuximab is a possible independent risk factor for TLI on a multivariate analysis. However, given the very small numbers of patients using targeted biologic agents, we found that concurrent cetuximab or nimotuzumab was not significantly correlated with TLI in our study.

Emami et al. [31] first reported that a dose of 60 Gy to 1/3 of the brain would cause a 5% risk of radiation-induced brain necrosis within 5 years. However, this study was based on literature reviews before 1991 (predating 3-dimensional conformal radiotherapy and IMRT), and this estimation is likely out-of-date in the era of IMRT. In 2010, Lawrence et al. [32] predicted incidences of 5 and 10% radiation brain necrosis to occur at doses of 72 Gy and 90 Gy in 2-Gy fractions, suggesting that a small volume of the brain could tolerate a higher dose.

This study presents a large data set used to assess the relationship among various treatment factors and TLI development in T4 NPC patients after IMRT. The advantages of our study include the use of standardized chemo-radiotherapy regimens, homogenous patient cohorts, and consistency in the TL contouring with one oncologist. These advantages help us minimize surveillance bias and focus on the dosimetric parameters. In multivariate competing risk regression model, TL V20 and D1cc were found to be predictive factors of TLI after IMRT and D1cc was considered as the most predictive. It was generally believed that TLI occurrence was related to a small volume of high-dose radiation, although the tolerance dose was not exactly the same [12, 17–20]. D1cc acted as a predictor of TLI, which is consistent with the conclusions of most studies mentioned above [12, 17, 18]. Among these reports, D1cc < 58 Gy, D1cc < 62.83 Gy and D1cc < 62.8 ± 2.2 Gy were found to be the tolerance doses of TL. However, these data seem to be conservative to ensure adequate tumor coverage for T4 NPC. In the previously published literature, patients with T1-T4 NPC were included, and for the T4 patients, the differentiation might not be good. The dose limit of the TL mentioned above is mainly calculated for TD 5/5 (radiation dose that could cause a 5% risk of complication in 5 years after irradiation) and the risk of TLI was analyzed without using death as a competing risk event, which could lead to an overestimation of the risk of developing TLI. According to clinical experience and actual research, the dose tolerance of the TL is higher than expected. After considering death a competing risk event among the 506 T4 NPC patients, under the premise of without comproming tumor coverage, the 5-year cumulative incidence of TLI was 13.2%, which was within the acceptable limits. In this nested case-control study, the 5-year cumulative incidence of TLI in the group with D1cc ≤71.14 Gy was 13.2%, which was much lower than the 5-year cumulative incidence of TLI in the group with D1cc > 71.14 Gy (62.2%, *P* < 0.001). Considering the balance of tumor coverage and TLI occurrence, we proposed a dose limit of D1cc ≤71.14Gy. A study involving 1887 NPC patients [16] demonstrated that the risk of TLI dramatically increased at a dose ≥70 Gy and that a dose of no more than 70 Gy was relatively safe. In a retrospective study involving 749 NPC patients, D0.5 cc < 73.66 Gy was found to be helpful in reducing the incidence of TLI [19]. Considering the treatment outcome, Ng et al. [33] proposed that Dmax ≤72 Gy is a new dose constraint for the TL. These results are similar to our results that D1cc ≤71.14 Gy could be a useful dose constraint.

Previous studies have shown that the primary prevention strategy for TLI is to avoid high dose delivery to the TL during IMRT. However, in this study, V20 was found to be another independent predictor of TLI occurrence, which might be regarded as a strange dose constraint for TLI. Su et al. [13] reported in their study involving 259 NPC patients treated with IMRT that V40 is highly predictive of TLI. As the dosimetric parameters V20 and V40 were highly correlated with each other (Spearman rank correlation coefficient of 0.832, *P* < 0.001), V20 acted as a predictor of TLI, which is consistent with the results reported by Su et al. [13]. In addition to high-dose radiation, low-dose radiation also plays important roles in the occurrence of TLI. Animal experiments [34, 35] suggested that exposing large volumes of adjacent normal tissues to lower-dose radiation could decrease the tolerance to toxicity when a small volume was exposed to a higher dose. When the brain is irradiated, oligodendrocytes are damaged, and when exposed to a large volume of lower-dose radiation, oligodendrocyte progenitor cells lose their proliferative ability. The damaged oligodendrocytes cannot be promptly renewed and replaced, affecting the repair of normal brain tissues that are exposed to high doses and ultimately leading to brain injury [36]. Potentially, a large volume of surrounding brain tissue exposed to low-dose radiation may have effects on the cellular environment and microvasculature that could increase the likelihood of brain injury. Although IMRT optimizes the dose distribution in the tumors, high dose to TLs overlapped with tumors seems to be inevitable in T4 NPC. Furthermore, the TLs receive a large volume of low- to moderate-dose irradiation, thus leading to the occurrence of TLI.

Target under-dosage due to OAR constraints appears to be correlated with an increased risk of local failure. The volume of under-dosage (< 66.5 Gy), even with a very small GTV volume (< 3.4 cm^3^), was highly detrimental to local control [33]. So, the importance of an adequate tumor coverage should be kept in mind. For T4 NPC, under the premise of tumor coverage, patients with D1cc ≤71.14 Gy and V20 ≤ 42.22 cc had significantly lower risks and cumulative incidences of TLI than those with D1cc > 71.14 Gy and V20 > 42.22 cc. These results suggested that D1cc ≤71.14 Gy and V20 ≤ 42.22 cc could be useful dose constraints of the TL without obviously interfering with the tumor coverage. In this study, the maximum point dose was not found to be the best predictor of TLI. Only the previously determined maximum dose constraint was inadequate for preventing TLI without considering the shape of the DVH. Efforts should also be made to limit a small volume of high-dose radiation and a large volume of low-dose radiation.

As a case-control study, it is possible that these results were affected by patient selection bias. Due to the small number of events available, the statistical ability was limited. In addition, various TL contouring methods could result in different dosimetric parameters [23]. A contouring guideline is needed to ensure that dosimetric parameters are comparable and reduce inter-institutional differences. Notably, different TPS may have effects on the dosimetric parameters. Our results were based on single-center data, and further validation through multicenter cooperation and consistency in the TL delineation are needed.

## Conclusions

TL D1cc and V20 are predictive of TLI after IMRT in T4 NPC. These parameters should be considered as the first and second priorities of dose constraints of the TL. Our results suggest that restricting D1cc ≤71.14 Gy and V20 ≤ 42.22 cc during IMRT optimization could help significantly reduce the occurrence of TLI in T4 NPC patients without obviously compromising the tumor coverage. To confirm our proposal, prospective studies based on these dose-volume constraints are needed in the future.

## Abbreviations

2D-RT: Two-dimensional radiotherapy
AJCC: American Joint Committee on Cancer
AUC: Area under the curve
CI: Confidence interval
CTV: Clinical target volume
DVH: Dose-volume-histogram
GTV: Gross tumor volume
IMRT: Intensity-modulated radiotherapy
NPC: Nasopharyngeal carcinoma
OAR: Organ at risk
OTT: Overall treatment time
PTV: Planning target volume
ROC: Receiver operating characteristic
RTOG: Radiation Therapy Oncology Group
sHR: Subdistribution hazard ratio
SIB: Simultaneous integrated boost
T1WI: T1-weighted image
T2WI: T2-weighted image
TL: Temporal lobe
TLI: Temporal lobe injury
TPS: Treatment planning system

## References

[1] Torre LA, Bray F, Siegel RL, Ferlay J, Lortet-Tieulent J, Jemal A (2015). Global cancer statistics, 2012. CA Cancer J Clin.

[2] Kam MK, Teo PM, Chau RM, Cheung KY, Choi PH, Kwan WH (2004). Treatment of nasopharyngeal carcinoma with intensity-modulated radiotherapy: the Hong Kong experience. Int J Radiat Oncol Biol Phys.

[3] Wolden SL, Chen WC, Pfister DG, Kraus DH, Berry SL, Zelefsky MJ (2006). Intensity-modulated radiation therapy (IMRT) for nasopharynx cancer: update of the memorial Sloan-Kettering experience. Int J Radiat Oncol Biol Phys.

[4] Tham IW, Hee SW, Yeo RM, Salleh PB, Lee J, Tan TW (2009). Treatment of nasopharyngeal carcinoma using intensity-modulated radiotherapy-the national cancer Centre Singapore experience. Int J Radiat Oncol Biol Phys.

[5] Su SF, Han F, Zhao C, Chen CY, Xiao WW, Li JX (2012). Long-term outcomes of early-stage nasopharyngeal carcinoma patients treated with intensity-modulated radiotherapy alone. Int J Radiat Oncol Biol Phys.

[6] Xiao WW, Huang SM, Han F, Wu SX, Lu LX, Lin CG (2011). Local control, survival, and late toxicities of locally advanced nasopharyngeal carcinoma treated by simultaneous modulated accelerated radiotherapy combined with cisplatin concurrent chemotherapy: long-term results of a phase 2 study. Cancer.

[7] Sun X, Su S, Chen C, Han F, Zhao C, Xiao W (2014). Long-term outcomes of intensity-modulated radiotherapy for 868 patients with nasopharyngeal carcinoma: an analysis of survival and treatment toxicities. Radiother Oncol.

[8] Wu LR, Liu YT, Jiang N, Fan YX, Wen J, Huang SF (2017). Ten-year survival outcomes for patients with nasopharyngeal carcinoma receiving intensity-modulated radiotherapy: an analysis of 614 patients from a single center. Oral Oncol.

[9] Lee AW, Kwong DL, Leung SF, Tung SY, Sze WM, Sham JS (2002). Factors affecting risk of symptomatic temporal lobe necrosis: significance of fractional dose and treatment time. Int J Radiat Oncol Biol Phys.

[10] Tang Y, Luo D, Rong X, Shi X, Peng Y (2012). Psychological disorders, cognitive dysfunction and quality of life in nasopharyngeal carcinoma patients with radiation-induced brain injury. PLoS One.

[11] Zhou GQ, Yu XL, Chen M, Guo R, Lei Y, Sun Y (2013). Radiation-induced temporal lobe injury for nasopharyngeal carcinoma: a comparison of intensity-modulated radiotherapy and conventional two-dimensional radiotherapy. PLoS One.

[12] Su SF, Huang Y, Xiao WW, Huang SM, Han F, Xie CM (2012). Clinical and dosimetric characteristics of temporal lobe injury following intensity modulated radiotherapy of nasopharyngeal carcinoma. Radiother Oncol.

[13] Su SF, Huang SM, Han F, Huang Y, Chen CY, Xiao WW (2013). Analysis of dosimetric factors associated with temporal lobe necrosis (TLN) in patients with nasopharyngeal carcinoma (NPC) after intensity modulated radiotherapy. Radiat Oncol.

[14] Sun Y, Zhou GQ, Qi ZY, Huang SM, Liu LZ, Li L (2013). Radiation-induced temporal lobe injury after intensity modulated radiotherapy in nasopharyngeal carcinoma patients: a dose-volume-outcome analysis. BMC Cancer.

[15] Zeng L, Tian YM, Sun XM, Chen CY, Han F, Xiao WW (2014). Late toxicities after intensity-modulated radiotherapy for nasopharyngeal carcinoma: patient and treatment-related risk factors. Br J Cancer.

[16] Zhou X, Ou X, Xu T, Wang X, Shen C, Ding J (2014). Effect of dosimetric factors on occurrence and volume of temporal lobe necrosis following intensity modulated radiation therapy for nasopharyngeal carcinoma: a case-control study. Int J Radiat Oncol Biol Phys.

[17] Zeng L, Huang SM, Tian YM, Sun XM, Han F, Lu TX (2015). Normal tissue complication probability model for radiation-induced temporal lobe injury after intensity-modulated radiation therapy for nasopharyngeal carcinoma. Radiology.

[18] Kong C, Zhu XZ, Lee TF, Feng PB, Xu JH, Qian PD (2016). LASSO-based NTCP model for radiation-induced temporal lobe injury developing after intensity-modulated radiotherapy of nasopharyngeal carcinoma. Sci Rep.

[19] Miao Y, Ou X, Wang J, Wang X, He X, Shen C (2017). Development and validation of a model for temporal lobe necrosis based on 749 nasopharyngeal carcinoma patients following IMRT. Int J Radiat Oncol Biol Phys.

[20] Feng M, Huang Y, Fan X, Xu P, Lang J, Wang D (2018). Prognostic variables for temporal lobe injury after intensity modulated-radiotherapy of nasopharyngeal carcinoma. Cancer Med.

[21] Koller MT, Schaer B, Wolbers M, Sticherling C, Bucher HC, Osswald S (2008). Death without prior appropriate implantable cardioverter-defibrillator therapy: a competing risk study. Circulation.

[22] Wang YX, King AD, Zhou H, Leung SF, Abrigo J, Chan YL (2009). Evolution of radiation-induced brain injury: MR imaging-based study. Radiology.

[23] Sun Y, Yu XL, Luo W, Lee AW, Wee JT, Lee N (2014). Recommendation for a contouring method and atlas of organs at risk in nasopharyngeal carcinoma patients receiving intensity-modulated radiotherapy. Radiother Oncol.

[24] Kong F, Ying H, Du C, Huang S, Zhou JJ, Hu C (2014). Effectiveness and toxicities of intensity-modulated radiation therapy for patients with T4 nasopharyngeal carcinoma. PLoS One.

[25] Cao CN, Luo JW, Gao L, Yi JL, Huang XD, Wang K (2013). Clinical outcomes and patterns of failure after intensity-modulated radiotherapy for T4 nasopharyngeal carcinoma. Oral Oncol.

[26] Cao CN, Luo JW, Gao L, Yi JL, Huang XD, Wang K (2015). Update report of T4 classification nasopharyngeal carcinoma after intensity-modulated radiotherapy: an analysis of survival and treatment toxicities. Oral Oncol.

[27] Luo Y, Gao Y, Yang G, Lang J (2016). Clinical outcome and prognostic factors of intensity-modulated radiotherapy for T4 stage nasopharyngeal carcinoma. Biomed Res Int.

[28] Lee AW, Foo W, Chappell R, Fowler JF, Sze WM, Poon YF (1998). Effect of time, dose, and fractionation on temporal lobe necrosis following radiotherapy for nasopharyngeal carcinoma. Int J Radiat Oncol Biol Phys.

[29] Lee AW, Ng WT, Hung WM, Choi CW, Tung R, Ling YH (2009). Major late toxicities after conformal radiotherapy for nasopharyngeal carcinoma—patient-and treatment-related risk factors. Int J Radiat Oncol Biol Phys.

[30] Ruben JD, Dally M, Bailey M, Smith R, McLean CA, Fedele P (2006). Cerebral radiation necrosis: incidence, outcomes, and risk factors with emphasis on radiation parameters and chemotherapy. Int J Radiat Oncol Biol Phys.

[31] Emami B, Lyman J, Brown A, Cola L, Goitein M, Munzenrider JE (1991). Tolerance of normal tissue to therapeutic irradiation. Int J Radiat Oncol Biol Phys.

[32] Lawrence YR, Li XA, el Naqa I, Hahn CA, Marks LB, Merchant TE (2010). Radiation dose-volume effects in the brain. Int J Radiat Oncol Biol Phys.

[33] Ng WT, Lee MC, Chang AT, Chan OS, Chan LL, Cheung FY (2014). The impact of dosimetric inadequacy on treatment outcome of nasopharyngeal carcinoma with IMRT. Oral Oncol.

[34] Bijl HP, van Luijk P, Coppes RP, Schippers JM, Konings AW, van der Kogel AJ (2006). Influence of adjacent low-dose fields on tolerance to high doses of protons in rat cervical spinal cord. Int J Radiat Oncol Biol Phys.

[35] van Luijk P, Faber H, Schippers JM, Brandenburg S, Langendijk JA, Meertens H (2009). Bath and shower effects in the rat parotid gland explain increased relative risk of parotid gland dysfunction after intensity-modulated radiotherapy. Int J Radiat Oncol Biol Phys.

[36] Chari DM, Huang WL, Blakemore WF (2003). Dysfunctional oligodendrocyte progenitor cell (OPC) populations may inhibit repopulation of OPC depleted tissue. J Neurosci Res.

